# Impact of Weight Reduction on Thyroid Function and Nonalcoholic Fatty Liver among Egyptian Adolescents with Obesity

**DOI:** 10.1155/2022/7738328

**Published:** 2022-03-29

**Authors:** Mona Karem Amin, Ahmed Ibrahim Ali, Hesham Elsayed

**Affiliations:** ^1^Pediatrics Department, Faculty of Medicine, Suez Canal University, Ismailia, Egypt; ^2^Pediatrics Department, Ministry of Health, Mansoura, Egypt

## Abstract

**Background:**

The prevalence of childhood obesity has been increasing worldwide. This may explain the emergence of nonalcoholic fatty liver as the leading cause of liver disease. Several previous studies have addressed the association between thyroid function and nonalcoholic fatty liver disease.

**Objectives:**

To study the impact of weight reduction through lifestyle modifications in adolescents with obesity.

**Methods:**

A prospective cohort study was done on 61 adolescents with obesity. Patients were evaluated at the first visit by the full history, clinical examination, and investigations (thyroid profile, lipid profile, liver function tests, HbA1c, and liver ultrasonography) as basal information. The intervention program included a dietary program, increasing physical activity, and decreasing sedentary activity. A postintervention evaluation was done at the end of six months which included anthropometric measures, laboratory results, and ultrasonographic estimation.

**Results:**

It was shown that the mean BMI of the participants had significantly decreased after lifestyle modification from (32.05 ± 3.36 kg/m2) to (28.1 ± 2.77 kg/m2) (*P* < 0.001). It also showed that the percentage of studied adolescents with elevated TSH decreased from 47.5% to 19.7% after the weight reduction program. Improvement was also achieved in the lipid profile and liver functions. The percentage of studied adolescents with ultrasound appearance of NAFLD decreased from 31.1% to 26.2% after weight reduction.

**Conclusions:**

Lifestyle modification positively influences the metabolic derangement in obesity without medical treatment. ΔTSH is a significant predictor of the change in BMI z-score. It is also possible that hepatic steatosis affects thyroid function rather than the other way around.

## 1. Introduction

Childhood obesity is considered a worldwide health problem. It has been increasing steady all over the world [1].

Nonalcoholic fatty liver disease (NAFLD) is now the most common form of chronic liver disease in childhood [2]. The real prevalence in the pediatric population is still unknown and variable. Obesity is considered the leading cause of NAFLD [3]. Although, a plethora of pharmacological treatment currently under trial for hepatic steatosis, nonpharmacological option in the form of lifestyle modification, is still the cornerstone in the management of such cases [4].

Obesity also affects hypothalamic-pituitary-thyroid (HTP) axis directly or indirectly leading to alterations in thyroid function tests [5]. Thyroid changes may be subclinical or overt. These thyroid changes become normalized after weight loss [5, 6].

Additionally, studies have declared the association between thyroid function and NAFLD [7–10].

Therefore, this study was done to clarify the effect of weight reduction through short-term lifestyle modifications program (6 months), including the changes in thyroid functions, lipid profile, and fatty liver parameters in obese adolescents.

### 1.1. Patients and Methods

A prospective cohort study was done on 61 patients of obese adolescents (10–18 years old). Patients were evaluated at first visit as basal information. Evaluation included the full history, clinical examination stressing on the presence of acanthosis nigricans (AN), and investigations (thyroid profile, lipid profile, liver function tests, HbA1c, and liver ultrasonography).

Weight reduction, family focused education was applied. The main components were dietary program, increasing physical activity, and decreasing sedentary activity. It was adapted from the WHO, ADA, and British Nutrition Foundation weight loss programs. Our endpoint was after six months. Frequent visits and interviews were done to maintain and ensure commitment of the family with our target patient.

At the end of six months, the final reevaluation was recorded including anthropometric measures, laboratory results, and abdominal ultrasonography.

Obesity was defined according to the BMI z-score
Overweight: BMI-for-age greater than one standard deviation above the WHO Growth Reference median and
Obesity is greater than 2 standard deviations above the WHO Growth Reference median, and to facilitate the improvement monitoring, obesity was divided as follows:
    Obese I: BMI is greater than 2 SD but less than +3 SD
    Obese II: BMI is greater than or equal +3 SD

Subclinical hypothyroidism is defined as elevated TSH levels with normal free T4 levels. TSH is considered high if more than 5 mU/L.

The fatty liver was diagnosed depending on the ultrasound findings by skillful, experienced radiologists.

Data were enrolled as spreadsheet using Microsoft Excel; then, all statistical analyses were done using the Statistical Package for Social Science version 20 (SPSS Inc., Chicago, IL, USA).

## 2. Results

The study was done on 61 adolescents with obesity. Their mean age was (13.56 ± 1.77) years with range between 10.5 and 17 years. Both genders were equally presented (Table 1).

### 2.1. Clinically

The mean of both BMI and BMI z-score of the participants had significantly decreased after intervention (28.1 ± 2.77 kg/m^2^, 2.34 ± 0.59, respectively) (*P* < 0.001). Accordingly, obesity grades were noted to be improved significantly after six months (*P* < 0.002).

Improvement was also reflected on the elevated blood pressure. It decreased from 13.2% to 4.9% after the weight reduction program. However, there was no statistically significant difference in the incidence of acanthosis nigricans (AN) (*P*=0.82). Only 1 from 15 participants showed the improvement of AN.

### 2.2. On the Laboratory Base

T4 and TSH improvement were significant (*P*=0.009, <0.001, respectively). The elevated TSH dropped within normal range in 17 adolescents. Additionally, lipid profile and liver functions were significantly reduced after commitment to lose weight, including HbA1c, TGs, cholesterol, LDL, and ALT levels (Table 2).

### 2.3. Radiologically

Ultrasound appearance of NAFLD was present in 31.1% of the sample before applying weight reduction program. Only 3 patients showed radiological improvement later at the end point of assessment.

It was found that the change in BMI z-score and the change in TSH values were significantly correlated with all the laboratory variables.  Table 3 provides the correlation between different clinical and laboratory measures.

Multivariable linear regression analysis was used to assess predictors of change in BMI among participants (Table 4). We found that ΔTSH was positively associated with change in BMI among participants, where for every 10 m IU/L increase in ΔTSH, there is an increase in ΔBMI by 5.28 kg/m^2^ (*P* < 0.001).

## 3. Discussion

It was found that the overall incidence of obesity grades among the studied participants had significantly decreased after applying the weight reduction program. A statistically significant reduction in the mean BMI was achieved within 6 months.

Studies were done based on lifestyle modification within variable duration showing successful outcomes. A German study was performed on 68 children with nonsyndromic obesity. The “Obeldicks” was the intervention program used. It was successful in reduction of their BMI z-scores by at least 0.1 [11]. In USA, lifestyle modifications were investigated in obese children with NAFLD; the study revealed a significant decrease in both bodyweight and BMI z-score throughout the weight loss intervals [12].

In the current study, the success was not only for the reduction of BMI but also in the comorbidities. While 13.2% of the studied adolescents had elevated arterial blood pressure (ABP) before the weight reduction program, only 4.9% still had elevated ABP by the end of the study. Many studies demonstrated the association between ABP and obesity. They showed high prevalence among the studied participants [12–16].

Kaltenbach et al. in Germany reported that the mean SBP and the mean DBP were 126.6 ± 11.8 and 77.1 ± 9.5 mmHg, respectively, among obese children [10]. However, our study showed lower values; the mean SBP and the mean DBP were 107.52 ± 11.34 and 62.59 ± 10.88 mmHg, respectively. The difference may be related to the lower sample size.

Moreover, lipid profile, ALT, and A1c were significantly correlated with the change in BMI. The results reflected the effect of weight reduction on decreasing the comorbidities. The same findings were reported in many studies dealing with weight loss through lifestyle modifications [12, 17]. These findings may reflect an important distinction between efficacy and effectiveness of weight loss in pediatric obesity.

Acanthosis nigricans (AN) was prevalent among our participants (> three quarters). AN screening is a simple clinical sign of insulin resistance (IR) [18–20]. It also reflects the increased risk for comorbidities and metabolic syndrome [21].

Notably, in our study, 3 out of the 61 participants showed regressed ultrasound features of NAFLD. The improvement of steatosis was reported in other studies [4]. Hepatic steatosis improved as a result of 5% bodyweight loss, and in general, the NAFLD activity score (NAS) improved with more than 7% weight reduction [22]. Relying on liver biopsies, another prospective study showed that lifestyle modifications causing weight loss of 10% were associated not only with improvement of both steatosis and fibrosis but with their resolution within 52 weeks [17].

The initial assessment of NAFLD is an integration of clinical, laboratory, and imaging evaluations [23]. Additionally, hepatic steatosis is still detected by ultrasonography when fatty hepatic infiltration is as low as 20–30% [24]. Accordingly, in the follow-up, the improvement is suspected earlier biochemically and histologically and then by ultrasound, as regression of hepatic steatosis occurred together with ALT improvement [24–26]. In the current study, ALT levels were decreased and correlated with the improvement of BMI, reflecting the decrease in hepatocellular inflammation and apoptosis and consequently NAFLD [27]. It seems that improvement may occur but is still partial yet ultrasonographically undetected. This may need longer follow-up or introduction of a therapeutic agent as prescribed by Negi et al. [28]. Many drugs are now on the pipeline trying to offer a solution for NAFLD.

EASL–EASD–EASO Clinical Practice Guidelines stated that “Diet and physical activity improve steatosis and hepatic inflammation in pediatric NAFLD, but no beneficial effects on fibrosis have ever been demonstrated” and then added “No safe drug treatment has proven effective on fibrosis in pediatric NAFLD” [24].

In the current study, thyroid dysfunction was documented and obviously much improved after weight reduction measures. It was shown in different studies, recent and earlier, that hyperthyrotropinemia is common among obese patients [9, 29–31]. Some studies also revealed the improvement of TSH following weight loss without additional treatment, [8, 32] while others like Reinehr and Andler's reported significant decrease in T3 and T4 without significant change in TSH levels with weight reduction [11].

The adolescents with elevated TSH (>5 mU/L) in the current study were 47.5%. This percentage decreased to 11.5% after weight loss. In addition, the mean TSH level of all the participants decreased from 5.24 to 3.95 mU/L after the weight reduction program, and this result was statistically significant (*P* < 0.05).

Theories had been generated to illustrate the thyroid dysfunction in obese patients [33]. It was explained upon the level of adipocytokines, mainly leptin, released from the adipocytes. Leptin affects thyroid centrally through the activation of thyrotropin-releasing hormone [34, 35] and, peripherally, by promoting the conversion of T4 to T3 [36]. However, TSH also increase the leptin production which depends on the adiposity mass [37]. Elevated TSH may also trigger low-grade chronic inflammation [33].

The current study reported that the effect of the change not only in BMI z-score but also in TSH levels was statistically significant and correlated with the different variables. The results highlight the vital role of both weight reduction and thyroid interplay in the management of obese patients. There is a complex and mutual relationship between the thyroid dysfunction and the adiposity [33].

In weight loss, change of body composition reduces the fat mass and reduction of the inflammatory process and cytokines and hence the leptin levels, leading to the improvement of thyroid function [32, 33].

Our linear regression analysis confirmed the BMI and TSH tight bond. It showed that for every 10 m IU/L increase in ΔTSH, there is an increase in ΔBMI by 5.28 kg/m^2^ and vice versa.

### 3.1. Limitation of the Study

This was a cohort study with a small sample size and time-limited interval. NAFLD was diagnosed radiologically by ultrasound as being present or not. No hepatic steatosis score was used like that described by Shannon et al. [38]. Additionally, grading of AN was not included and was recorded as being present or absent and that may lead to *β*-error.

## 4. Conclusion

Weight reduction without medical treatment can improve the metabolic derangements in obese adolescents.

Thyroid function improvement is an early sign for weight reduction success.

Long-term follow-up is needed especially with NAFLD. Improvement may need further weight reduction or just an extended time on the healthy lifestyle.

## Figures and Tables

**Table 1:** Demographic characteristics of the participants.

Variables	(*n* = 61)

Age (years)	
Mean ± SD	13.56 ± 1.77
Median (range)	13.5 (10.5–17)
Gender, *n* (%)	
Male	30 (49.2)
Female	31 (50.8)

**Table 2:** Comparison of clinical, laboratory, and radiological variables of the studied participants before and after weight reduction measures.

Variables	Before mean ± SD	After mean ± SD	*P* value^a^
BMI	32.05 ± 3.36	28.1 ± 2.77	**<0.001** ^*∗*^^**a**^
BMI (z-score)	2.96 ± 0.52	2.34 ± 0.59	**<0.001** ^*∗*^^**a**^
Obesity grades			
Overweight	0 (0)	16 (26.2)	**0.002** ^*∗*^^**b**^
Obese I	39 (63.9)	35 (57.4)	
Obese II	22 (36.1)	10 (16.4)	
Acanthosis nigricans			
Absent	47 (77)	48 (78.7)	0.82^b^
Present	14 (23)	13 (21.3)	
Blood pressure, *n* (%)			
Normal	53 (86.8)	58 (95.1)	**0.002** ^*∗*^^**b**^
Elevated	8 (13.2)	3 (4.9)	
Systolic BP (mmHg)	107.52 ± 11.34	101.68 ± 9.71	**0.008** ^*∗*^
Diastolic BP (mmHg)	62.59 ± 10.88	59.09 ± 10.36	**0.001** ^*∗*^
Serum T4 (ug/dl)	11.0 ± 1.65	10.4 ± 1.59	**0.009** ^*∗*^^**a**^
TSH (mU/L)	5.2 ± 2.86	3.9 ± 1.59	**<0.001** ^*∗*^^**a**^
TSH, *n* (%)			
Normal	32 (52.5)	49 (80.3)	**0.011** ^*∗*^^b^
Elevated	29 (47.5)	12 (19.7)	
HbA1c (%)	4.7 ± 0.37	4.5 ± 0.29	**<0.001** ^*∗*^^**a**^
Lipid profile			
Triglyceride (mg/dl)	136.4 ± 26.6	115.6 ± 18.4	**<0.001** ^*∗*^^**a**^
Total cholesterol (mg/dl)	160.3 ± 20.1	145.3 ± 13.6	**<0.001** ^*∗*^^**a**^
LDL (mg/dl)	113.5 ± 20.6	99.2 ± 14.7	**<0.001** ^*∗*^^**a**^
HDL (mg/dl)	47.4 ± 4.9	47.3 ± 5.7	0.975^a^
ALT (IU/L)	41.8 ± 18.4	36.0 ± 10.2	**<0.001** ^*∗*^^**a**^
US for the fatty liver, *n* (%)			
Absent	42 (68.9)	45 (73.8)	0.54^b^
Present	19 (31.1)	16 (26.2)	

^a^ *P* values are based on the paired *t*-test. Statistical significance at *P* < 0.05. ^b^*P* values are based on McNemar's test. Statistical significance at *P* < 0.05. Bold values represent the significant improvement after weight reduction measures.

**Table 3:** Correlation between different clinical and laboratory variables.

Variables	ΔBMI (z-score)	ΔTSH	ΔT_4_	ΔHbA1c	ΔTG	Δcholesterol	ΔHDL	ΔLDL	ΔALT
Age	−0.161	−0.090	−0.113	−0.115	−**0.405**^*∗*^	−**0.403**^*∗*^	0.111	−**0.299**^*∗*^	−0.219
ΔBMI (z-score)	—	**0.610** ^*∗*^	**0.353** ^*∗*^	**0.373** ^*∗*^	**0.343** ^*∗*^	**0.487** ^*∗*^	−**0.330**^*∗*^	**0.407** ^*∗*^	**0.416** ^*∗*^
ΔTSH	—	—	**0.561** ^*∗*^	**0.511** ^*∗*^	**0.493** ^*∗*^	**0.588** ^*∗*^	−**0.273**^*∗*^	**0.536** ^*∗*^	**0.446** ^*∗*^
ΔT_4_	—	—	—	**0.484** ^*∗*^	**0.339** ^*∗*^	0.168	−0.238	**0.302** ^*∗*^	**0.439** ^*∗*^
ΔHbA1c	—	—	—	—	**0.387** ^*∗*^	**0.455** ^*∗*^	−0.158	**0.347** ^*∗*^	**0.403** ^*∗*^
ΔTG	—	—	—	—	—	**0.786** ^*∗*^	−0.115	**0.792** ^*∗*^	**0.579** ^*∗*^
Δcholesterol	—	—	—	—	—	—	−0.134	**0.787** ^*∗*^	**0.463** ^*∗*^
ΔHDL	—	—	—	—	—	—	—	−**0.323**^*∗*^	−**0.468**^*∗*^
ΔLDL	—	—	—	—	—	—	—	—	**0.659** ^*∗*^

*P* values are based on Pearson's correlation coefficient. ^*∗*^Statistical significance at *P* < 0.05. Δ, the overall change in the given value. Bold represents the significant improvement after weight reduction measures.

**Table 4:** Multivariable linear regression analysis of determinants of change in BMI among participants.

Predictors	Unstandardized coefficients		Standardized coefficients	*P* value
	*B*	Std. error	Beta	
Constant	−0.116−	0.444	—	0.794
Age	−0.028−	0.032	−0.092−	0.372
Gender (female)	0.194	0.121	0.180	0.114
ΔTSH	0.168	0.036	0.528	**<0.001** ^*∗*^

ANOVA <0.001, *R*^2^ **=** 0.411. ^*∗*^Statistical significance at *P* < 0.05.

## Data Availability

The datasets used and/or analyzed during the current study are available from the corresponding author upon request.
